# Last Word on Viewpoint: Using V̇o_2max_ as a marker of training status in athletes–can we do better?

**DOI:** 10.1152/japplphysiol.00238.2022

**Published:** 2022-07-01

**Authors:** Tim Podlogar, Peter Leo, James Spragg

**Affiliations:** ^1^School of Sport, Exercise and Rehabilitation Sciences, University of Birmingham, Birmingham, United Kingdom; ^2^Faculty of Health Sciences, University of Primorska, Izola, Slovenia; ^3^Human Performance Centre, Ljubljana, Slovenia; ^4^Division of Performance Physiology & Prevention, Department Sports Science, University of Innsbruck, Innsbruck, Austria; ^5^Health through Physical Activity, Lifestyle and Sports (HPALS), Research Centre, Faculty of Health Sciences, University of Cape Town, Cape Town, South Africa

First, we would like to thank our peers for sharing their views (1) on our Viewpoint (2). There appears to be some consensus that V̇o_2max_ as such is an insufficient descriptor of the training status.

We agree that no laboratory-based measure will ever perfectly describe training status nor performance. Indeed, had this been the case, there would be no need for competition!

Ultimately, the best descriptor of performance is performance itself. However, there are cases when we may have no, or insufficient, competition/performance data to allow accurate participant classification. In these cases, we stand by our view that if one parameter is to be used, on balance, CP/CS and its corresponding W’ offer the best insight into training status. We fully encourage researchers to describe participants as comprehensively as possible, encompassing all available measures, including performance/competition data, to assist the readership in understanding training status. Our objective in proposing CP/CS over and above V̇o_2max_ was to raise the floor and not the ceiling in terms of participant classification. CP/CS represents a physiology descriptor that is *1*) relatively practical to measure and *2*) is a better predictor of performance than currently widespread approaches (3).

A concern expressed in some letters related to methodological issues with the CP/CS concept. Namely, the impact of trial duration and mathematical modeling on CP/CS estimates. To address this briefly; all trials should fall within the severe exercise intensity domain ensuring attainment of V̇o_2max_. In practice, only trials between 2 and 15 min are suitable. Multiple trials (3+) are recommended to avoid skewness in modeling in the case of imperfect pacing. A possible exception is in athletes habituated to CP/CS prediction trials; here two trials may be sufficient (4). If these recommendations are followed, the applied mathematical model has a negligible effect on the CP/CS estimation (<2%) (5). However, choosing the model which results in the best fit is arguably the best practice. Strict adherence to this methodology has been shown to produce CP/CS estimates with a small coefficient of variance (0.8% and 4.6% for CP and W’, respectively) (6).

As with any physiological measure, environmental factors will affect CP/CS estimates, therefore, as a good research practice, researchers should report detailed information about the testing procedures, making the subsequent interpretation of the data easier, and improving experimental/intervention reproducibility.

We acknowledge a current paucity of CP/CS data in published literature. However, it is evident that if researchers were to adopt the proposed approach, more data would quickly become available, allowing normative values to be determined. We firmly believe that the historical use of V̇o_2max_ is not a strong enough justification to not replace it with a measure that better reflects training status.

To conclude, we continue to argue that we should stop classifying research participants based solely on V̇o_2max_. We propose that researchers adopt as many available measures as possible when classifying participants to give the readership a better understanding of the applicability of research findings. However, when minimal descriptors are available, we continue to advocate for the use of the CP/CS concept.

## DISCLOSURES

No conflicts of interest, financial or otherwise, are declared by the authors.

## AUTHOR CONTRIBUTIONS

T.P., P.L., and J.S. drafted manuscript; T.P., P.L., and J.S. edited and revised manuscript; T.P., P.L., and J.S. approved final version of manuscript.
